# Seasonal climatic niche-switching migration in the Nearctic-Neotropical Rufous Hummingbird (*Selasphorus rufus*)

**DOI:** 10.1371/journal.pone.0334958

**Published:** 2025-12-04

**Authors:** Edson A. Alvarez-Alvarez, R. Carlos Almazán-Núñez, Felipe A. Toro-Cardona, L. Gerardo Herrera M, María José Tolsá-García, Oscar Talavera-Mendoza, Martha Lopezaraiza-Mikel

**Affiliations:** 1 Doctorado en Recursos Naturales y Ecología, Facultad de Ecología Marina, Universidad Autónoma de Guerrero, Acapulco, Guerrero, México; 2 Departamento de Fortalecimiento a la Investigación e Innovación Tecnológica, Consejo de Ciencia, Tecnología e Innovación del Estado de Guerrero, Chilpancingo de los Bravo, Guerrero, México; 3 Laboratorio de Ecología y Biogeografía de la Conservación, Facultad de Ciencias Químico-Biológicas, Universidad Autónoma de Guerrero, Chilpancingo de los Bravo, Guerrero, México; 4 Grupo de Ecología y Evolución de Vertebrados, Instituto de Biología, Facultad de Ciencias Exactas y Naturales, Universidad de Antioquia, Medellín, Colombia; 5 Instituto de Biología, Estación de Biología Chamela, Universidad Nacional Autónoma de México, Chamela, Jalisco, México; 6 Laboratorio de Virología, Facultad de Medicina Veterinaria y Zootecnia, Universidad Nacional Autónoma de México, Ciudad de México, México; 7 Escuela Superior de Ciencias de la Tierra, Universidad Autónoma de Guerrero, Taxco de Alarcón, Guerrero, México; 8 Laboratorio Nacional de Análisis y Síntesis Ecológica, Escuela Superior de Desarrollo Sustentable, Universidad Autónoma de Guerrero, Técpan de Galeana, Guerrero, México; Indian Institute of Science, INDIA

## Abstract

Migratory birds inhabit diverse geographic regions during their reproductive and wintering periods; however, information regarding the climatic conditions that enable migratory birds, particularly hummingbirds, to thrive in these regions is limited. This study aimed to characterize the geographic distribution and seasonal climatic niche of the Nearctic–Neotropical Rufous Hummingbird (*Selasphorus rufus*)—classified as near threatened—across its reproductive and wintering areas in North America. We obtained monthly occurrence records for the Rufous Hummingbird during its reproductive (May to June) and wintering (November to January) periods. We generated seasonal ecological niche models to reconstruct the climatic space occupied by the Rufous Hummingbird during these periods and evaluated climatic differences through niche similarity analysis. Our results indicate that the Rufous Hummingbird is a longitudinally migratory species that engages in niche-switching, occupying distinct climatic conditions in its reproductive and wintering areas across Mexico and the eastern United States. Changes in the climatic niche of the Rufous Hummingbird may be linked to its adaptive plasticity in response to varying climatic conditions, competition for resources, and recent climate change. These findings have biogeographic, evolutionary, and conservation implications for the Rufous Hummingbird and other migratory birds with similar movement patterns.

## Introduction

In North America, avian migration involves the movement of millions of individuals from at least 386 bird species. These migratory species travel from reproductive (Nearctic region) to non-reproductive or wintering (Neotropical region) areas [1]. The primary factors driving avian migration are food resources and climatic conditions [2,3]. However, seasonal variations in biotic resources, such as food availability for many migratory bird species, remain largely unknown. This gap exists because mechanistic information—such as physiological, biophysical, or functional traits of species (i.e., Eltonian niche)—is generated at fine scales and is unlikely to be included in species distribution models (SDMs) or ecological niche models (ENMs) executed at coarser scales [4]. In contrast, climate-based correlative models are fundamental tools for studying migratory bird species [5,6], particularly regarding the climatic conditions influencing their movements between reproductive and wintering areas.

ENMs have improved our understanding of seasonal climatic variation and its overlap during avian migratory movements [7]. Some migratory birds, termed “niche trackers”, track similar climatic conditions in their reproductive and wintering areas, indicating that they exhibit more similar seasonal climatic niches [8–10]. In contrast, “niche switchers” experience different climatic conditions due to their broader climatic tolerance, which results in more distinct seasonal climatic niches [5,6,11]. Therefore, examining climatic conditions during both reproductive and wintering periods can help determine seasonal ecological niches and assess the recent and potential impacts of climate change on avian migration [12].

Hummingbirds, despite being among the smallest migratory birds, are surprisingly capable of covering long distances during migration. Existing ENM studies on hummingbirds have primarily focused on evolutionary and anthropogenic issues such as climate and land-use change, providing insights into speciation, diversification processes, and conservation strategies [13–15]. However, there is a lack of studies examining other factors such as environmental conditions influencing hummingbird migration. To date, only the migration patterns of the Broad-tailed Hummingbird (*Selasphorus platycercus*) have been documented in relation to seasonal climatic variations [6,16]. For the 13 other latitudinally migratory hummingbird species, the climatic conditions shaping their distributions over time remain largely unknown.

**Fig 1:**
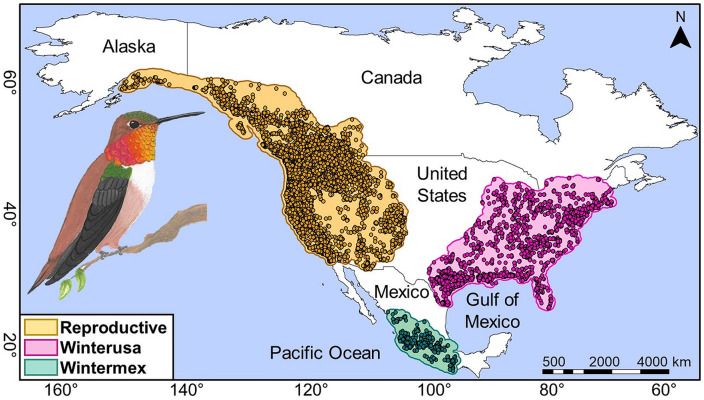
Occurrence records in the reproductive and wintering areas (winterusa and wintermex) of the Rufous Hummingbird. Polygons surrounding the occurrence records represent accessible areas. The image of the Rufous Hummingbird was obtained from Almazán-Núñez et al. [42].

The Rufous Hummingbird (*Selasphorus rufus*) is a Nearctic–Neotropical migratory species that breeds in the northwestern USA, Canada, and southeastern Alaska from May to June [17,18, S1 Fig]. While most populations migrate, some appear to remain in their breeding areas year-round (S1 Fig). Fall migration typically occurs from late July to October, whereas spring migration takes place from late February to April [18]. From November to January, Rufous Hummingbirds winter in southern, central, and western Mexico, as well as the southeastern USA [17–19, S1 Fig]. Several populations that migrate to the southeastern USA make secondary movements within the wintering areas of Mexico in response to resource availability and environmental conditions (i.e., intratropical migration) [20–22]. During migration, Rufous Hummingbirds make stopovers between their breeding and wintering areas (see Fig 1), which can last from several days to weeks. These stopovers allow them to recover energy, rebuild muscle, and replenish fat reserves [17,18]. To support this, Rufous Hummingbirds feed on nectar from at least 66 plant species along their migratory routes [17,19,23,24].

**Fig 2:**
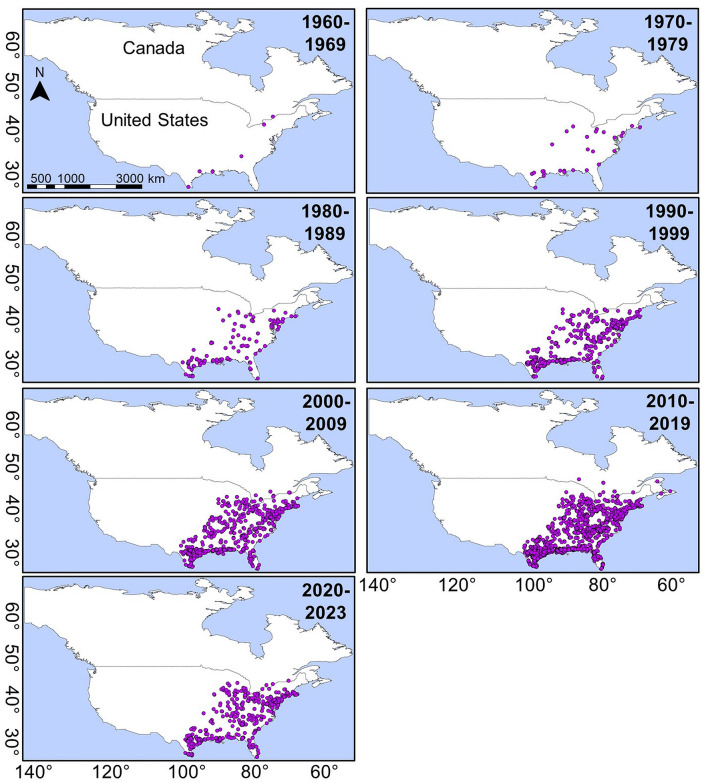
Occurrence records of the Rufous Hummingbird. Records are presented in 10-year periods from the first sightings during its winter migration toward southeastern, eastern, and northeastern USA and southeastern Canada.

Most studies on Rufous Hummingbirds have focused on their feeding behavior, genetic divergence, migratory connectivity, and demographic patterns [25–28]. However, there is limited information on the climatic conditions that shape their ecological niches in both the reproductive and wintering areas. Although the Rufous Hummingbird’s winter migration was previously understood to occur primarily in a latitudinal direction toward Mexico and the southeastern USA, consistent evidence of longitudinal migration toward the eastern-northeastern USA and southeastern Canada has emerged over the past 40 years (Fig 2). While there is no definitive hypothesis explaining these longitudinal movements, they may be linked to the species’ adaptive plasticity in response to resource competition and recent climate changes in its wintering areas [17,29]. Similar longitudinal migratory behavior has also been observed in the Broad-tailed Hummingbird, which migrates toward southeastern USA [6].

Notably, the Rufous Hummingbird is classified as a “near-threatened” species [30]. Estimates suggest that its breeding populations have declined sharply by 43%–65%, largely due to climate change and habitat loss [31,32]. Understanding the climatic conditions that enable the persistence of this migratory hummingbird is crucial for developing effective conservation strategies [6].

In this study, we characterized the Rufous Hummingbird’s geographic distribution and seasonal climatic niche in both its reproductive and wintering areas. Given the climatic variability across its geographic range and the documented seasonal climatic niche-switching behavior of other migratory hummingbird species (i.e., the Broad-tailed Hummingbird), we assessed whether the Rufous Hummingbird shifts its climatic niche between its migration areas [6]. Specifically, we predicted that (1) the climatic niche of the Mexican wintering area (located in the Neotropical region) would differ from those of the USA’s reproductive and wintering areas (located in the Nearctic region), due to greater climatic variation between these biogeographic regions; and (2) that climatic niches in the USA’s reproductive and wintering areas would exhibit greater similarity, given their comparable environmental conditions. This study underscores the role of climate in shaping the migratory behavior, geographic distribution, ecological niche, and conservation of the Rufous Hummingbird amid ongoing climate change.

## Materials and methods

### Occurrence data

We obtained monthly occurrence records of the Rufous Hummingbird from the Global Biodiversity Information Facility database (S1 Table) and the Atlas of the Birds of Mexico, which compiles distributional data from various natural history museums worldwide [33]. Additionally, we consulted the regional bird collection database of the Laboratorio de Ecología y Biogeografía de la Conservación, Universidad Autónoma de Guerrero (LEBCO-UAGro). We divided these occurrences according to the reproductive and wintering seasons and areas of this hummingbird. Reproductive (May to June) and wintering (November to January) areas from 1960 to 2023 correspond to periods during which populations of this hummingbird have been documented to remain relatively stable in each of these areas [17,19]. The wintering area was divided into two regions. The first wintering area, located in the USA and Canada (hereafter referred to as “winterusa”), is predominantly distributed across the Southeast USA conifer savannas, the Central USA forest-grassland transition, the Southern Great Lakes forests, the Piney Woods, and the Appalachian-Blue Ridge forests, ecoregions situated within the Nearctic region. In contrast, the second wintering area, located in Mexico (hereafter referred to as “wintermex”), is primarily distributed across the Trans-Mexican Volcanic Belt pine-oak forests, the Sierra Madre Occidental pine-oak forests, the Sierra Madre del Sur pine-oak forests, the Central Mexican matorral, the Balsas dry forests, and the Sinaloan dry forests—ecoregions within the Neotropical region [34,35]. These two wintering areas are situated in ecoregions characterized by generally distinct physiographic, ecological, and climatic conditions [34,36,37].

We generated a database of geographic coordinates, dates, and the countries where the Rufous Hummingbird is distributed. We then, used a geographic information system (ArcMap v10.3) to map those occurrences and detect and remove doubtful, erroneous, or poorly georeferenced records (points projected in oceans or at extreme edges of the known distributional range). Furthermore, occurrence records associated with resident populations—those present year-round in specific portions of the reproductive and wintering areas—were excluded, as these populations do not undertake long-distance migratory movements. Given that ecological niche characterization requires unique occurrence records per location, we removed duplicate monthly records within each seasonal area using the *ntbox* package [38] in R Statistical Software (v4.1.0) [39]. To further reduce bias from spatial autocorrelation due to overrepresented occurrence data, we applied a five-kilometer spatial thinning [40] using the *ntbox* package [38]. This process resulted in 9,309 spatially unique occurrences for the reproductive area, 2,040 for winterusa, and 405 for wintermex (Fig 1). We consider this spatial thinning threshold adequate *i)* for reducing record overrepresentation in areas with high data concentration (i.e., reproductive and winterusa areas), while still being moderate enough to retain sufficient data in regions with lower occurrence density (i.e., wintermex), *ii)* to maintain consistency with the resolution of the bioclimatic variables employed (see Climate Data section) and, *iii)* because the floral resources exploited by the rufous hummingbirds are typically distributed in patches located at relatively short distances [18,41].

### Delimitation of accessible areas

Accessible areas represent the environments the Rufous Hummingbird has explored, reflecting its movement opportunities and constraints. We delineated accessible areas for the species’ reproductive and wintering ranges using occurrence records consistently distributed across the terrestrial ecoregions of North America [34]. Defining accessible areas based on biogeographic entities or ecoregions is widely accepted due to its efficiency, accuracy, and strong performance in ecological niche and species distribution models [43,44]. Additionally, to account for the Rufous Hummingbird’s movements, we added a 60-km buffer around selected ecoregions within each of the three seasonal areas (reproductive and wintering). This buffer allowed for the inclusion of occurrence records located near the boundaries of each seasonal area (Fig 1).

### Climatic data

We used seven monthly climate variables from the WorldClim database (v2.1) [45], provided in raster format with a spatial resolution of 2.5 min (~ 4.5 km^2^). The selected variables included minimum temperature (ºC, MinT), maximum temperature (ºC, MaxT), average temperature (ºC, AverT), precipitation (mm, Prec), solar radiation (kJ m^-2^ day^-1^, SolRad), wind speed (m s^-1^, WindS), and water vapor pressure (kPa, Wvp) for the reproductive (May to June) and wintering (November to January) periods. All climate variables were included in a Spearman correlation analysis using the *ntbox* package [38] to minimize issues arising from multicollinearity. On basis of this analysis, we selected four variables with correlation coefficients below 0.85: maximum temperature, precipitation, solar radiation, and wind speed. These variables were used to generate geographic distribution models for each seasonal area of the Rufous Hummingbird. These variables have been previously documented to influence seasonal distribution patterns in migratory *Selasphorus* species and play a critical ecological role in the survival of this hummingbird [6,32]. These four climatic variables were clipped to the reproductive and wintering areas. To better characterize the environmental conditions in each seasonal area, we estimated accumulated precipitation by summing the monthly rasters (i.e., May + June for the reproductive area and November + December + January for the wintering areas). For the other three climatic variables, we estimated their average values for seasonal and reproductive areas.

### Similarity analysis of seasonal climatic niches

Climatic similarity between the reproductive and wintering areas was analyzed using Schoener’s D index, implemented via the *ecospat* package [45,46] in R Statistical Software (v4.1.0) [39]. Schoener’s D index ranges from 0 (different niches) to 1 (similar niches) [46]. This niche similarity test employs principal component analysis of environmental variables (PCA-env) as the ordination method and applies relative occurrence densities for each season, using a kernel density function. These densities were then used to calculate the relative frequencies of climatic conditions in each seasonal area, which were projected onto the climatic space defined by the first two axes of the PCA-env. The observed D values were then compared to a distribution of D values generated from null models. Specifically, the observed D metric was derived from 100 randomizations of occurrence density locations within the climatic space (PCA-env) of a seasonal area [46,47]. In this analysis, we hypothesized that the climatic conditions among the wintering and reproductive areas are more different than expected by the null model. To test the niche permutation hypothesis—which posits that the overlap of seasonal climatic niches is more different than expected by chance—we set the alternative parameter as “lower.” A *p*-value greater than 0.05 indicates that the observed overlap is not significantly lower than what would be expected if the niches were randomly distributed within the available environmental space.

### Analysis of seasonal climatic variability

The four climatic variables were independently evaluated using a Kruskal-Wallis test to assess climatic variability among the three seasonal areas occupied by the Rufous Hummingbird. A Wilcoxon *post-hoc* pairwise test was subsequently performed to identify which seasonal areas differed significantly in terms of climate. For this analysis, values for each climatic variable were extracted from the occurrence records of the Rufous Hummingbird in both reproductive and wintering areas. Seasonal climatic differences were considered significant when *p* ≤ 0.05 according to the Kruskal–Wallis and Wilcoxon *post-hoc* pairwise tests.

### Species distribution models

Distribution models for each reproductive and wintering area were generated using the Maximum Entropy algorithm (MaxEnt v3.4.1.) [48], implemented through the *kuenm* package [49] in R Statistical Software (v4.1.0) [39]. For model development, we randomly divided the species occurrences into two subsets: 80% for training and the remaining 20% for testing—thresholds commonly used in other hummingbird studies [6]. We incorporated three feature classes—linear (l), product (p), and quadratic (q)—and six combinations: “lq,” “q,” “p,” “qp,” “lqp,” and “lp.” Additionally, we tested different values of regularization multiplier to represent various complexity levels in the model and avoid overfitting. Therefore, we used values from 0.1 to 1 in 0.1 increments for strict models and values from 2 to 6 in 1-unit increments for more relaxed models [50]. The *kuenm* package combines these parameters to create candidate models for evaluation. These models are evaluated using statistical criteria, including the area under the receiver operating characteristic curve (partial ROC *p* ≤ 0.05; AUC ratio > 1), the Akaike information criterion (dAIC < 2), and predictive performance based on the omission rate (false-negative rate ≤ 5%) [49].

Once we obtained the final models (best evaluated) for each area, we transferred them between seasonal areas to assess whether the Rufous Hummingbird tracks or shifts its climatic preferences between reproductive and wintering regions. Each of the three seasonal areas served as a calibration area, where the model was trained using species occurrence data and subsequently projected onto another specific seasonal area. This approach was designed to assess climatic similarity across the geographical space of the Rufous Hummingbird’s seasonal areas. Model transfers included both no extrapolation and free extrapolation scenarios. No extrapolation models constrain predictions to environmental conditions within the range observed during model calibration, thereby limiting projections to analogous climatic spaces [49,51]. In contrast, free extrapolation is usually applied when a species has a wide physiological capacity that allows it to be present in different climatic conditions [52,53]. Thus, if the Rufous Hummingbird were a niche tracker, with the non-extrapolation model, we would expect to obtain distribution predictions from the calibration areas to the projection areas. Whereas, if the species is a niche switcher, the model with free extrapolation would identify the non-analogous climatic regions where the species is distributed.

To estimate the degree of extrapolation—or the presence of non-analogous climates—between seasonal areas, we used the multivariate environmental similarity surface (MESS), which identifies regions where different levels of climatic similarity are observed between calibration and transfer areas [51]. The MESS metric quantifies how environmentally similar each location is based on the most dissimilar variable relative to the reference conditions [51].

In both approaches (free extrapolation and no extrapolation), we performed 10 bootstrap replicates and selected the cloglog format to scale raw suitability values from 0 (non/low suitable) to 1 (highly suitable). Then we used the median of these replicates as the final model. Subsequently, to obtain the potential distribution of the Rufous Hummingbird in the three seasonal areas, we transformed the continuous output (suitability values) into presence-absence maps. To achieve this, we used the 10th percentile training presence threshold, which estimates the value (threshold) at which 10% of training records present the lowest predicted suitability values. This thresholding method helps minimize overprediction in the output maps [54].

### Ethics statement

The methods employed in this research do not require ethical approval, as the data were derived from publicly available information.

## Results

### Variability of seasonal climatic niches

**Fig 3:**
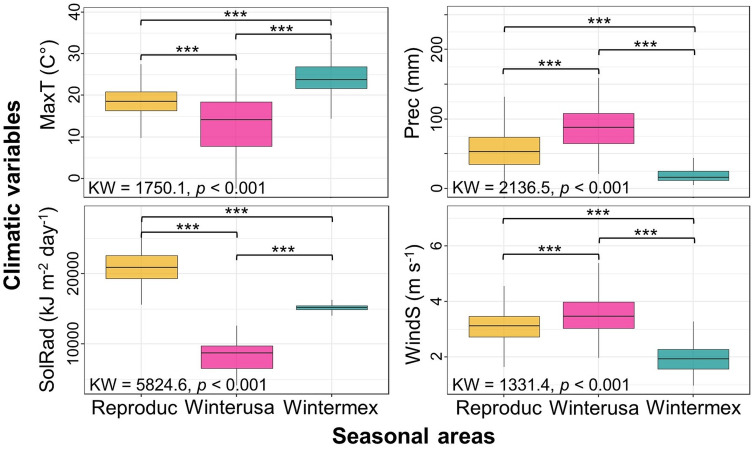
Climatic variables and their differences among the seasonal areas occupied by the Rufous Hummingbird. Climatic variables include maximum temperature (MaxT), precipitation (Prec), solar radiation (SolRad), and wind speed (WindS). Significant seasonal climatic differences are denoted as *p* < 0.001 (***) according to the Wilcoxon *post-hoc* pairwise test.

The two main components of PCA-env explained 76% of the total cumulative variance. Maximum temperature and wind speed were correlated with principal component (PC) 1, while precipitation and solar radiation were associated with PC2 (S2 Table and S2 Fig). This finding indicates that these environmental factors are grouped according to their relationship with different aspects of the climate. Furthermore, we found significant environmental variability among the three seasonal areas across four climatic variables, as determined by the Kruskal–Wallis and Wilcoxon *post-hoc* pairwise tests (*p* < 0.001). Solar radiation emerged as the most significantly different climatic variable (Fig 3). The Rufous Hummingbird occupied nearly 50% of the available environments in both the reproductive and wintering areas (S2 Fig).

### Similarity of seasonal climatic niches

**Fig 4:**
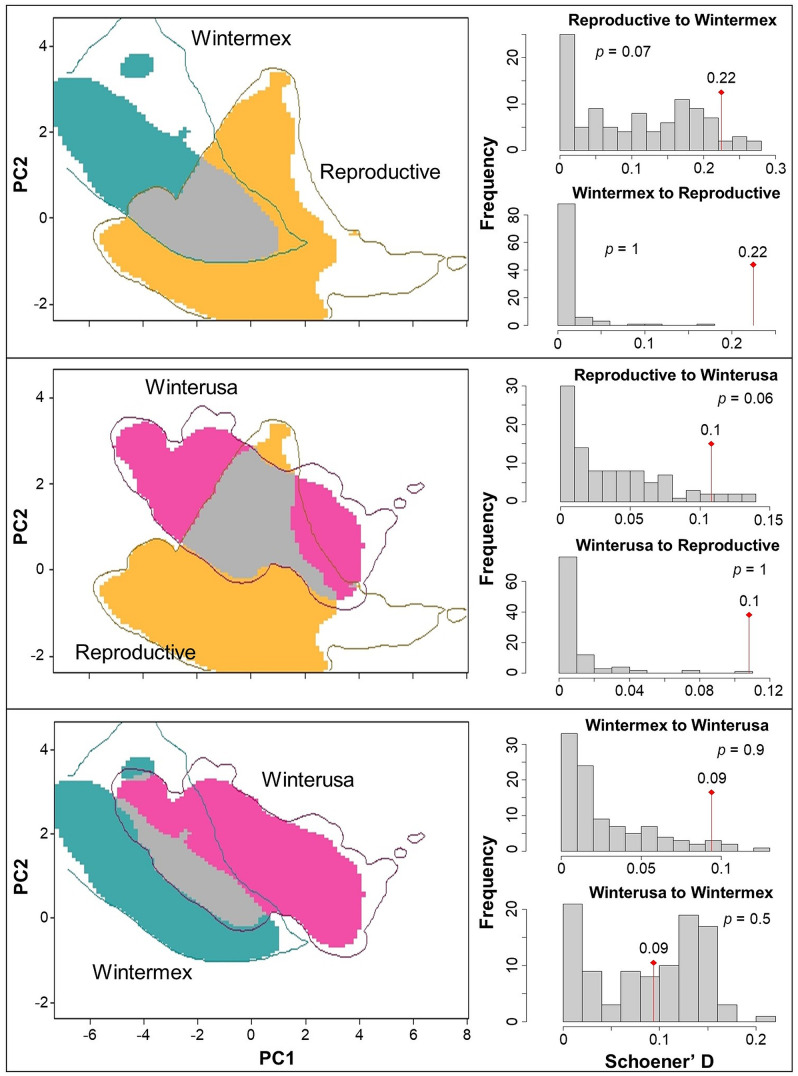
Niche similarity and significance among seasonal climatic niches of the Rufous Hummingbird. The orange, pink, and blue-green colors in the left panels indicate the occupied climatic conditions, while the gray color represents overlap among two seasonal climatic niches. Contour lines depict available climatic conditions. Histograms in the right panels show the distribution of Schoener’s D values (climatic niche similarity), comparing observed (red line) *versus* random (null model or gray bars) use of environmental space between seasonal areas.

We found low climatic similarity between all seasonal areas. The climatic niches of the reproductive and wintermex areas exhibited a 22% similarity (Schoener’s D = 0.22). The climatic niches of the reproductive and winterusa areas showed a 10% similarity (Schoener’s D = 0.1), while the winter climatic niches had a similarity value of 9% (Schoener’s D = 0.09). In all cases, the three seasonal climatic niches were not more different than expected by chance, given the available environmental space of each seasonal area (*p* > 0.05, Fig 4).

### Distribution in reproductive and wintering areas

**Fig 5:**
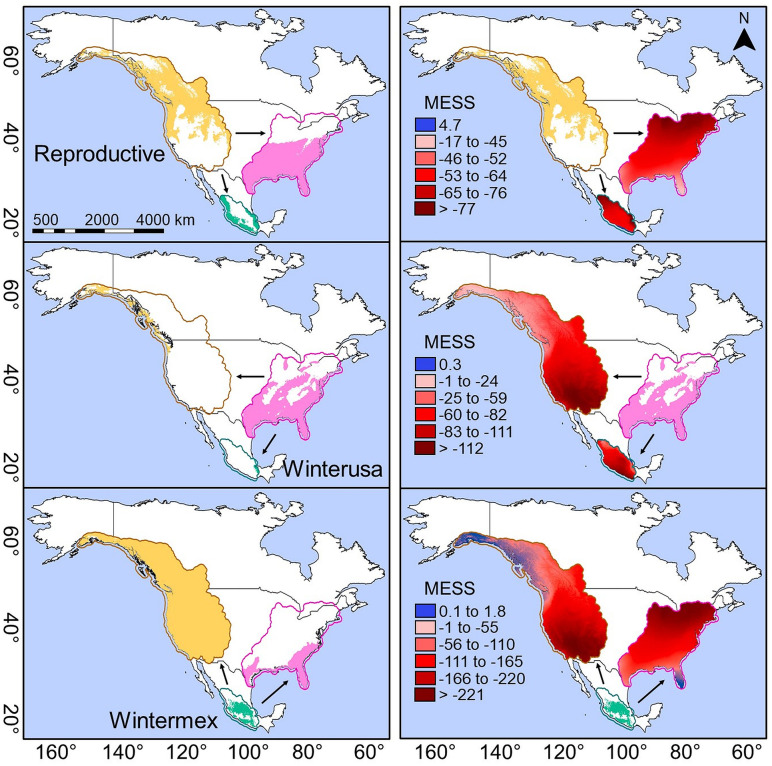
Geographic distribution models with free extrapolation (left panels) and MESS analysis (right panels). The orange, pink, and blue-green colors represent the seasonal geographic distribution of the Rufous Hummingbird. Polygons surrounding the seasonal distributions and MESS models denote accessible areas. Arrows indicate the direction of climatic condition transfer among the seasonal areas. Red areas with negative MESS scores highlight regions with non-analogous conditions between the calibration areas and the transferred areas.

Of the candidate models evaluated in the seasonal areas, only one model met the evaluation criteria suggested by *kuenm*. The distribution models exhibited low omission rates (< 0.5), indicating good predictive performance. Additionally, the models were statistically significant based on the AUC ratio (> 1) and dAIC (< 2) (Table 1). Overall, non-extrapolation distribution models revealed small portions of potential distribution among seasonal areas (reproductive to wintermex and *vice versa*, winterusa to wintermex, and *vice versa*; S3 Fig), which aligns with the low niche similarity values we found. Furthermore, these results are supported by the MESS analysis, which indicates that most of the area among the three seasons corresponds to non-analogous climates. In contrast, models that used free extrapolation across the three seasonal areas showed a wider proportion of predicted area (Fig 5). Models with free extrapolation predicted 97% of occurrence records for the Rufous Hummingbird in areas with analogous projections, compared to only 20% of records predicted by non-extrapolation models.

**Table 1:** Model parameters showing highest predictive accuracy for reproductive and wintering areas.

Seasonal area	Feature class	Regularization multiplier	dAIC	AUC ratio	Omission rate
Reproductive	qp	5	228612.86	1.02	0.04
Winterusa	qp	0.2	48560.49	1.1	0.01
Wintermex	qp	0.4	8198.31	1.09	0.04

*Parameters:* dAIC = Akaike information criterion, AUC = area under the partial ROC curve. *Feature class:* quadratic (q), product (p).

## Discussion

Our results suggest that the Rufous Hummingbird is a niche-switching species, occupying different climatic conditions in its reproductive and wintering areas. Seasonal climatic niche analysis revealed low overlap (9%–22%, highest between reproductive and wintermex areas) in the use of multivariate climatic space. This overlap was not significantly different from what would be expected by chance, given that the environmental conditions between each seasonal area are considerably different. This observation is also supported by the poor transfer of distribution models among seasonal areas when no extrapolation was applied. This pattern was especially evident in model transfers from wintermex to reproductive areas.

As hypothesized, the climatic niche of the Rufous Hummingbird at wintermex differed from those of the reproductive and winterusa areas. However, a lower degree of climatic niche similarity was observed between the latter two seasonal areas. As previously noted, avian migration is influenced by climatic availability, suggesting that migratory bird species can exhibit niche-tracking or niche-switching behavior [7]. We hypothesize that, similar to patterns observed in certain invasive species, an observed niche shift between different areas (in our case, seasonal areas) may be attributable to the species’ fundamental niche being broader than the environmental conditions available in their distribution ranges [55,56]. Consequently, the Rufous Hummingbird may occupy a larger portion of its fundamental niche during its migratory cycle—a “realized niche shift”—due to its physiological tolerance. This capacity enables the species to thrive across a wide range of environmental conditions (from subarctic to tropical climates, i.e., 1.2°C to 24.1°C) and ecological tolerances (from high mountain grasslands to dry, temperate, and humid tropical forests) throughout its range. Therefore, this adaptability suggests that the Rufous Hummingbird’s broad geographic range is shaped by the breadth of its fundamental niche, allowing it to colonize climatically diverse sites across both its reproductive and wintering areas [57]. This aspect has also been examined in the context of climate change and the recent distributional shifts observed in several migratory species in the USA and Europe [58].

The climatic differences among the three seasonal niches may be related to the migratory strategies or long-distance movements undertaken by the Rufous Hummingbird [28], which span large spatial areas and may drive seasonal climatic niche differentiation in the species. Specifically, migratory birds traveling longer distances are more likely to encounter greater climatic variation between their reproductive and wintering areas. This differentiation functions as a survival strategy, allowing species to adapt to distinct climatic conditions in both regions. Previous studies have shown that the niche-switching strategy is positively correlated with migratory distance in birds; that is, as migratory distance increases, the overlap between seasonal climatic niches tends to decrease [11,59,60].

In contrast, the environmental suitability for many migratory bird species is decreasing due to recent climate change; consequently, migratory birds search for new environmentally suitable sites based on their physiological tolerance ranges [12]. For instance, variations in temperature, precipitation, and humidity have been documented in the southeastern USA within the winterusa region due to recent climate change [29,61]. These non-analogous climatic modifications could lead to niche abandonment or the development of pioneer niches [62]. The latter may be occurring in the wintering areas occupied by the Rufous Hummingbird over the past 40 years in the northeastern USA and southeastern Canada (see Fig 2). Hence, the migratory behavior and geographic distribution of the Rufous Hummingbird may undergo shifts, potentially leading to the exploration and colonization of novel areas, changes in migratory routes, or winter movements toward the northeastern USA and southeastern Canada. Although this phenomenon of niche and/or geographic range expansion in breeding ranges due to recent climate change must be confirmed for the Rufous Hummingbird, other migratory birds in the USA and Europe have experienced similar changes, including Anna’s hummingbirds (*Calypte anna*) [58]. For example, Anna’s hummingbirds have been documented expanding their range into colder and more northerly regions, a shift attributed to factors such as urbanization and the increased availability of supplemental feeding. This range expansion underscores potential alterations in both the hummingbird species’ geographic distribution and migratory behavior [63].

Our results indicate that solar radiation is the most significantly different climatic variable affecting the seasonal geographic distribution and climatic niches of the Rufous Hummingbird, presumably playing a critical role in plant flowering, growth, reproduction, and photosynthesis [64–66]. The relationship of flower phenology and climate change has not yet been examined in the Rufous Hummingbird, but they could provide relevant insights into the Eltonian or functional niche (i.e., interactions between species and their resources) and the potential effects of recent climate change on the Grinnellian niche (i.e., tolerance ranges to environmental conditions) during its migratory cycle. This is important because ENMs typically do not incorporate mechanistic information regarding species’ physiological, biophysical, or functional traits (Eltonian niche) [4]. Additionally, the climatic variations—and the low niche overlap—between the two wintering areas are associated with solar radiation, which is higher in central Mexico than in the southeastern USA [67]. For instance, in wintermex, the Rufous Hummingbird inhabits temperatures ranging from 8.2°C to 24.1°C, with an average monthly precipitation of 19.7 mm. In contrast, in winterusa, temperatures range from 2.2°C to 13.3°C, with an average precipitation of 86.6 mm. The low overlap in the wintering climatic niches, largely driven by differences in solar radiation, indicates that the Rufous Hummingbird occupies climatically distinct wintering environments, which could eventually lead to the emergence of two ecologically distinct populations. This finding aligns with genetic differences observed in breeding populations of this migratory hummingbird [26]. Thus, solar radiation influences natural processes such as the water cycle, temperature, and plant phenology, all of which are related to climatic patterns that shape global biodiversity.

Another explanation for the low overlap between wintering areas is intratropical migration. After arriving in Mexico or the southeastern USA, Rufous Hummingbirds may undertake secondary movements in response to resource limitations, shifting toward the northeastern USA and southeastern Canada where conditions are more favorable. Such intratropical movements—secondary migrations within the non-breeding season—have been documented in other bird species, reflecting responses to changing resource availability and environmental conditions [21].

In contrast, the longitudinal migration of the Rufous Hummingbird warrants attention. This species typically migrates latitudinally toward southern Mexico and the southeastern USA [17,19]. However, over the past 40 years, some Rufous Hummingbirds have been recorded migrating longitudinally from the western to the eastern and northeastern USA, as well as to southeastern Canada. This pattern is evident in Rufous Hummingbird occurrence records (see Fig 2), and several hypotheses have been proposed to explain this migratory behavior.

One possibility is that longitudinal migration results from migratory detours that altered the species’ original route, as suggested by Hill et al. [68], who proposed a shift in wintering sites from Mexico to the southeastern USA. Another potential explanation is bi-coordinate navigation, which refers to the ability to identify both latitude and longitude. This phenomenon suggests that the Rufous Hummingbird may not only determine its latitudinal position but could also use geomagnetic information for longitudinal displacement, as has been observed in migratory white-crowned sparrows (Passerellidae) and Eurasian reed warblers (Acrocephalidae) in Europe and North America [69,70]. However, this hypothesis requires further investigation and should be explored in future spatial learning studies on the Rufous Hummingbird.

Our results also suggest that distinct climatic conditions at reproductive and wintering sites could eventually reproductively isolate populations of the Rufous Hummingbird and other migratory hummingbirds. For instance, ENMs have been used to propose the existence of genetically differentiated populations in migratory birds [11,71]. In the case of the Rufous Hummingbird, genetically distinct populations have been identified within its breeding range [26]. However, these subspecies or populations are not formally recognized, and to date, the Rufous Hummingbird is considered to lack any officially designated subspecies or differentiated populations [18,72]. Nevertheless, the Rufous Hummingbird’s combined latitudinal and longitudinal migratory patterns—particularly movements toward two distinct wintering grounds—could, over time, promote reproductive isolation and lead to the emergence of ecologically and genetically distinct populations. A similar process appears to have occurred in the recently described species split of the Giant Hummingbird (*Patagonia gigas*) with migratory and resident populations exhibiting genetic and morphological divergence [73].

As noted earlier, factors such as the search for food, the ability to track floral resources, environmental variability, and recent climate change may contribute to population isolation among Rufous Hummingbirds migrating to separate wintering grounds. Isotopic studies have even reported allopatric sexual segregation within the Mexican wintering areas of this species [27]. Genetic differences among Rufous Hummingbird breeding populations, attributed to isolation during the last glaciation have also been suggested [26]. Additionally, climate-related divergence has been documented in both migratory and resident populations of the Broad-tailed Hummingbird [74]. The Anna’s Hummingbird and Allen’s Hummingbird (*Selasphorus sasin*) provide other examples, with populations divided into resident and migratory groups [75–78]. Future research on the Rufous Hummingbird should investigate the genetic structure of both its reproductive and wintering populations.

Given current evidence, the segregation of individuals into two distinct wintering areas may facilitate reproductive isolation among populations. This isolation could arise through at least two non-mutually exclusive mechanisms: *i)* divergence along differential winter migratory routes, where reproductive dispersal is constrained by geographic distance, leading to a gradual shift in allele frequencies consistent with isolation by distance [79,80]; and *ii)* divergent selective pressures encountered in ecologically distinct wintering habitats—such as differences in climate, floral resources availability, interspecific competition—which may drive both ecological and reproductive divergence, as previously mentioned. This isolation could limit genetic exchange, potentially resulting from the species colonizing new habitats and becoming sedentary or from the development of distinct migratory routes, as has been observed in other migratory birds exhibiting partial migration [81]. However, key aspects such as bi-coordinate navigation, speciation, and partial migration in the Rufous Hummingbird remain understudied. These factors are crucial for understanding the ecological, biogeographic, and evolutionary implications of migration in this species.

## Conclusions

Our study reveals that climate significantly influences the migratory behavior, geographic distribution, and ecological niche of the Rufous Hummingbird. We found that this species exhibits seasonal climatic niche-switching behavior, primarily due to its adaptive plasticity in response to varying climatic conditions, competition for resources, and recent climate change. Understanding seasonal niche suitability is crucial, as climate change and habitat loss are dramatically altering the migratory behavior and distribution patterns of the Rufous Hummingbird’s breeding populations [31,32]. This suggests that anthropogenic threats, such as urban expansion, wildfires, and changes in climate and land use, should be considered in future research regarding the ecology, biogeography, and demography of the Rufous Hummingbird across its Mexican winter distribution range. Similarly, this declining pattern has also been documented for other endemic or resident hummingbird species in Mexico and their associated plant resources [13,82], which may have significant ecological impacts on pollination by hummingbirds. Our study emphasizes that climatic conditions are essential for addressing questions related to the physiology, biogeography, evolution, conservation, and anthropogenic challenges faced by migratory bird species in the context of changing climatic events. Finally, we suggest that future studies should address the concepts of niche permutation and tracking in the context of migratory or seasonally active species. For instance, if a species’ fundamental niche is composed by the climatic conditions that the species go through their different seasons, then the existence of niche permutation species must be questioned.

## Supporting information

S1 Table Citation data of the monthly occurrence records of the Rufous Hummingbird. Occurrence records were obtained on May 8, 2023, from the Global Biodiversity Information Facility database (https://www.gbif.org/).
(PDF)

S2 Table Loadings of climatic variables in the principal component analysis. (PDF)

S1 Fig Monthly occurrence records of the Rufous Hummingbird. The records illustrate the reproductive (May to June) and winter (November to January) months, as well as the spring (February to April) and fall (July to October) migrations.
(TIFF)

S2 Fig Principal component analysis of climatic variables and seasonal climatic niches of the Rufous Hummingbird. Climatic niches are represented by the first two axes of the PCA. The gray shading indicates the area with the highest occurrence record density in the climatic space. The dotted and solid lines represent 50% and 100% of the available climatic conditions for the Rufous Hummingbird, respectively. Climatic variables include maximum temperature (MaxT), minimum temperature (MinT), average temperature (AverT), precipitation (Prec), water vapor pressure (Wvp), solar radiation (SolRad), and wind speed (WindS).
(TIFF)

S3 Fig Non-extrapolation distribution models of seasonal areas of the Rufous Hummingbird. The orange, pink, and blue-green colors represent the seasonal geographic distribution of the Rufous Hummingbird. Polygons surrounding these seasonal distributions and suitability models denote accessible areas. Warmer colors in black boxes indicate regions with better-predicted conditions. The arrows indicate the transfer direction of climatic conditions in the seasonal areas.
(TIFF)
