# Supplementary material for: Seasonal climatic niche-switching migration in the Nearctic-Neotropical Rufous Hummingbird (Selasphorus rufus)
Source: PLoS One. 2025 Dec 4;20(12):e0334958. doi: 10.1371/journal.pone.0334958 (PMC12677473; doi:10.1371/journal.pone.0334958)
Supplement: S1 Table — Occurrence records were obtained on May 8, 2023, from the Global Biodiversity Information Facility database (https://www.gbif.org/). (PDF) [file pone.0334958.s001.pdf]

S1 Table. Citation data of the monthly occurrence records of the Rufous Hummingbird. Occurrence records were obtained on May 8, 2023, from the Global Biodiversity Information Facility database (https://www.gbif.org/)

Month	DOI
January	https://doi.org/10.15468/dl.vchzq4
February	https://doi.org/10.15468/dl.ysbahp
March	https://doi.org/10.15468/dl.ey9b6w
April	https://doi.org/10.15468/dl.89ukwr
May	https://doi.org/10.15468/dl.2psdhq
June	https://doi.org/10.15468/dl.a3pyxa
July	https://doi.org/10.15468/dl.28t7h4
August	https://doi.org/10.15468/dl.btc6r5
September	https://doi.org/10.15468/dl.rcrgvn
October	https://doi.org/10.15468/dl.takjnp
November	https://doi.org/10.15468/dl.5ydx9a
December	https://doi.org/10.15468/dl.wf9cjx
