# Supplementary material for: Seasonal climatic niche-switching migration in the Nearctic-Neotropical Rufous Hummingbird (Selasphorus rufus)
Source: PLoS One. 2025 Dec 4;20(12):e0334958. doi: 10.1371/journal.pone.0334958 (PMC12677473; doi:10.1371/journal.pone.0334958)
Supplement: S2 Table — (PDF) [file pone.0334958.s002.pdf]

S2 Table. Loadings of climatic variables in the principal component analysis.

Climatic variables	Principal component 1	Principal component 2
Precipitation	0.25	0.55
Water vapor pressure	-0.41	0.35
Solar radiation	-0.26	-0.60
Maximum temperature	-0.49	-0.20
Minimum temperature	-0.45	0.34
Average temperature	-0.48	0.23
Wind speed	0.18	0.07
