# Supplementary figures and images for: Seasonal climatic niche-switching migration in the Nearctic-Neotropical Rufous Hummingbird (Selasphorus rufus)
Source: PLoS One. 2025 Dec 4;20(12):e0334958. doi: 10.1371/journal.pone.0334958 (PMC12677473; doi:10.1371/journal.pone.0334958)

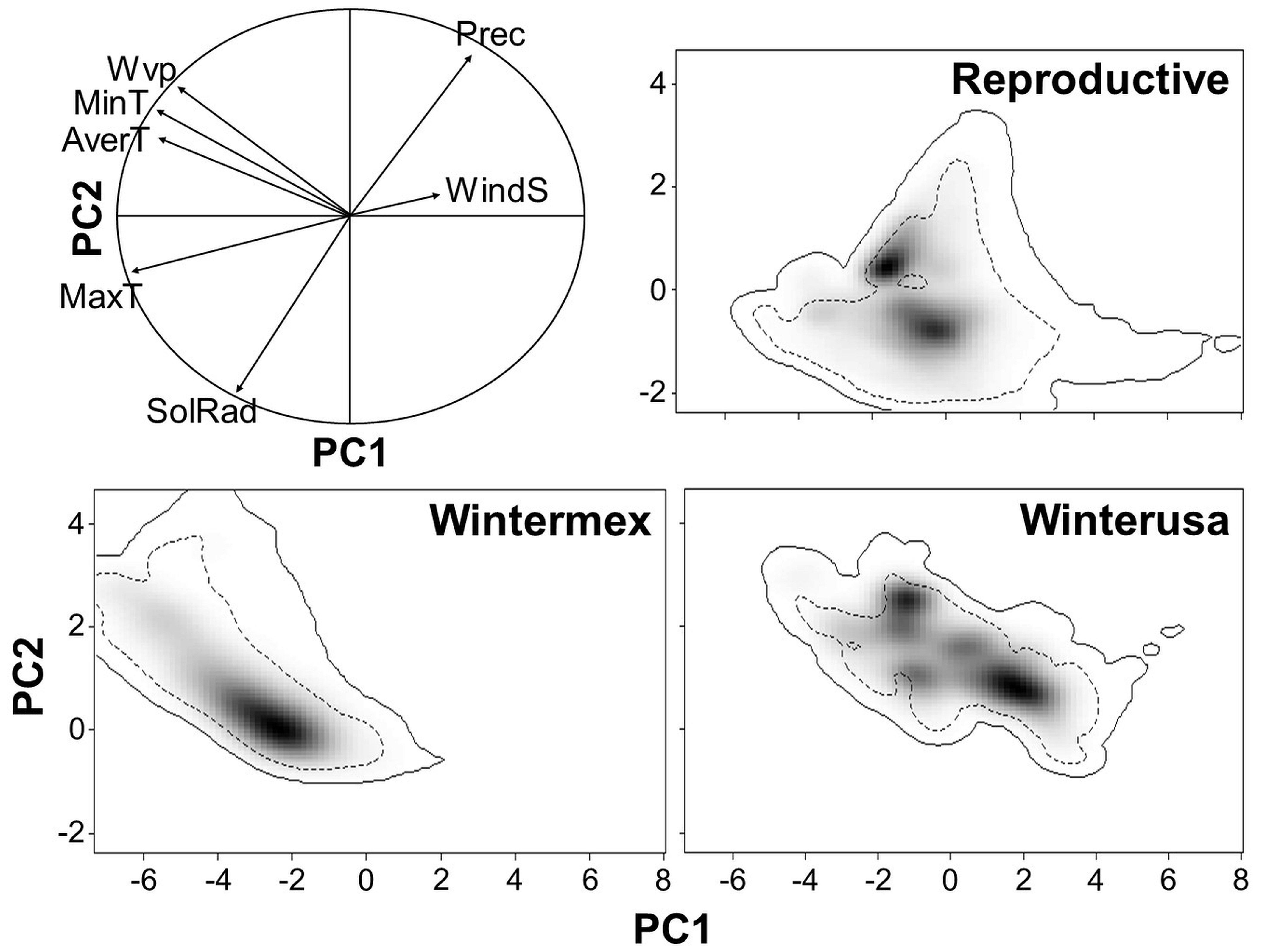

Supplement: S2 Fig — Climatic niches are represented by the first two axes of the PCA. The gray shading indicates the area with the highest occurrence record density in the climatic space. The dotted and solid lines represent 50% and 100% of the available climatic conditions for the Rufous Hummingbird, respectively. Climatic variables include maximum temperature (MaxT), minimum temperature (MinT), average temperature (AverT), precipitation (Prec), water vapor pressure (Wvp), solar radiation (SolRad), and wind speed (WindS). (TIFF) [file pone.0334958.s004.tif]

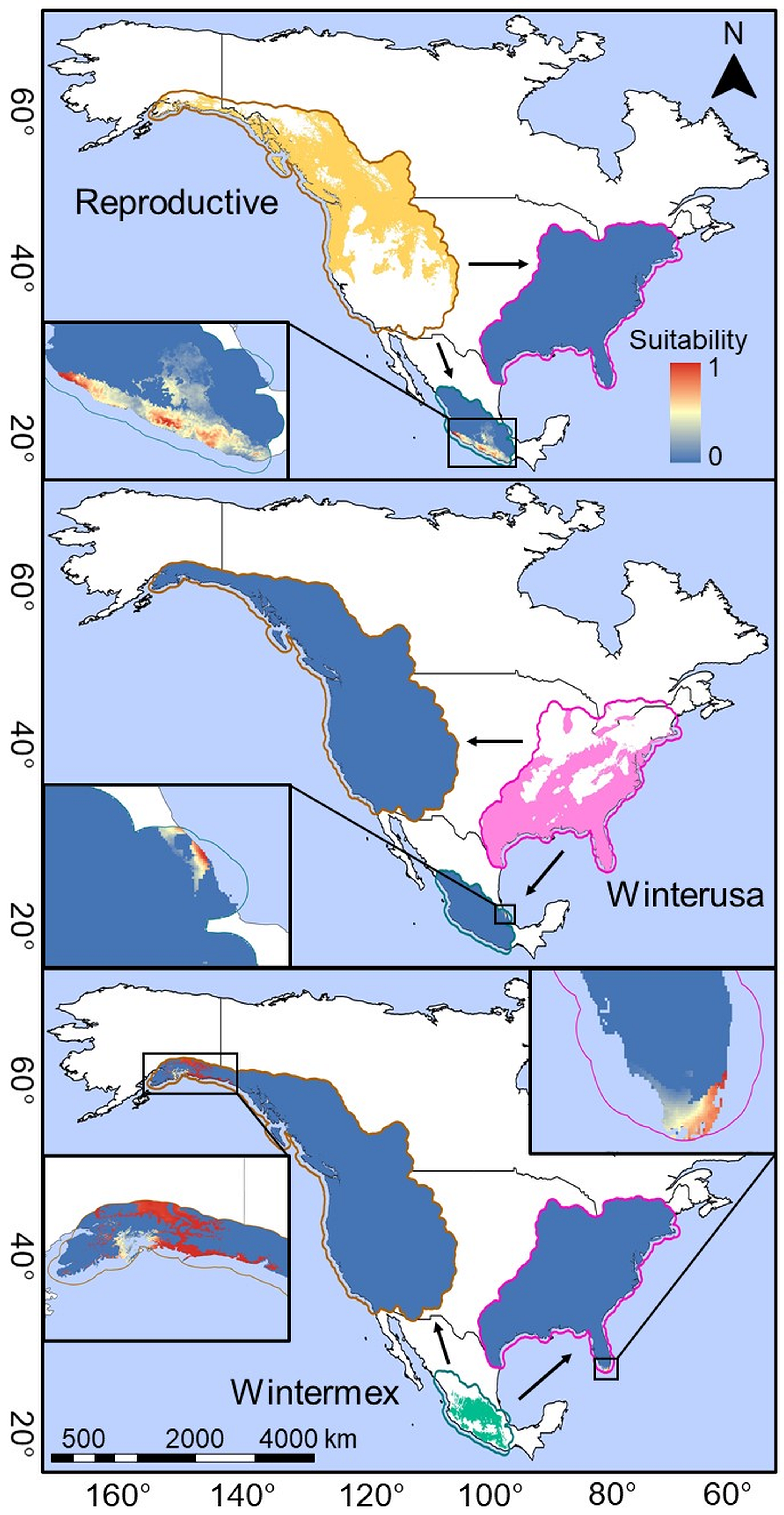

Supplement: S3 Fig — The orange, pink, and blue-green colors represent the seasonal geographic distribution of the Rufous Hummingbird. Polygons surrounding these seasonal distributions and suitability models denote accessible areas. Warmer colors in black boxes indicate regions with better-predicted conditions. The arrows indicate the transfer direction of climatic conditions in the seasonal areas. (TIFF) [file pone.0334958.s005.tif]
